# Quality of life and its predictive factors among women with obstetric fistula in Ethiopia: A cross-sectional study

**DOI:** 10.3389/fpubh.2022.987659

**Published:** 2022-10-28

**Authors:** Bekana Fekecha Hurissa, Zewdie Birhanu Koricha, Lelisa Sena Dadi

**Affiliations:** ^1^School of Midwifery, Institute of Health, Jimma University, Jimma, Ethiopia; ^2^Department of Health, Behavior, and Society, Faculty of Public Health, Jimma University, Jimma, Ethiopia; ^3^Department of Epidemiology, Faculty of Public Health, Jimma University, Jimma, Ethiopia

**Keywords:** obstetric fistula, predictors, quality of life, women with fistula, Ethiopia

## Abstract

**Objective:**

Living with obstetric fistulas is detrimental to the quality of life of women with fistulas. This study aimed to assess the quality of life and predictive factors among women with obstetric fistula in Ethiopia.

**Methods:**

A cross-sectional study was employed on consecutively selected 478 women. Linear regressions were used for data analysis.

**Results:**

The mean quality of life in physical, psychological, social, and environmental health domains and the overall quality of life were 40.59 ± 1.58, 38.10 ± 1.78, 29.59 ± 1.97, 34.21 ± 1.65, and 44.61 ± 3.99 respectively. Repair outcome without urinary inconsistence (β = 5.2; 95% CI = 0.72, 9.64), self-esteem (β = 1.3; 95% CI = 0.96, 1.57), negative attitude (β = 5.1; 95% CI = 1.86, 8.33), waiting treatment (β = −8.4; 95% CI = −15.54, −1.10), and low intention (β = 4.7; 95% CI = 1.52, 7.93) were predictors of the quality of life in physical domain. Repair outcome without urinary inconsistence (β = 5.9; 95% CI = 1.73, 9.99), self-esteem (β = 1.8; 95% CI = 1.47, 2.11), negative attitude (β = −6.4; 95% CI = −9.60, −3.25), fathers at primary school (β = 12.5; 95% CI = 0.08, 24.82), living only with parents (β = 4.9; 95% CI = 0.99, 8.90), time of care-seeking (β = −0.01, 95% CI = −0.02, −0.002), and duration lived with fistula (β = −5.4; 95% CI = −9.12, −1.68) were predictors of psychological domain. Dead birth (β = −5.2; 95% CI = −9.86, −0.51), self-esteem (β = 1.1; 95% CI = 0.72, 1.43), and living only with parents (β = 5.5; 95% CI = 0.30, 10.69), and living only with husband (β = 7.8; 95% CI = 2.01, 13.55) were predictors of social domain. Living in rural (β = −6; 95% CI = −9.22, −2.79), women at secondary school (β = 14.1; 95% CI = 3.67, 24.48), self-esteem (β = 1.3; 95% CI = 0.99, 1.55), negative attitude (β = −5.1; 95% CI = −7.97, −2.29) were predictors of quality of life in environmental domain. Repair outcome without urinary inconsistence (β = 8.3; 95% CI = 0.62, 16.02), self-esteem (β = 2.1; 95% CI = 1.34, 2.79), and living only with parents (β = 2.9; 95% CI = 1.06, 4.76) were significant predictors of the overall quality of life.

**Conclusions:**

The quality of life of women with obstetric fistula was low. Repair outcomes, self-esteem, negative attitudes, rural residence, living with parents, and time of care-seeking were significant predictors of quality of life. Urgent measures should be taken to address these factors to improve the quality of life of women with fistula.

## Introduction

Obstetric fistula is the presence of an unusual opening between a woman's vagina and the bladder or rectum through which her pee and/or feces persistently spill (1). Sometime recently in the 20th century, both urinary and rectal fistulas were common results of birth processes all over the world (2). Nevertheless, nowadays it is established in destitution, mainly influencing marginalized women who do not get quality obstetric care, with lower financial status, are not educated, are in the countryside, are with no antenatal care, and are hitched at more youthful ages (3). It has been a serious and debilitating complication of childbirth and maternal morbidity with an obliterating impact on a woman's life and remains the predominant cause of maternal morbidity within developing countries (4, 5). Women with obstetric fistulas remain chronic women and experience limits in their daily activities (6). Consistent spillage of pee and/or feces leads to gigantic physical, psychosocial, and financial injuries and most such women put themselves into suicidal ideations (7).

Quality of life has developed as a basic well-being component that broadens customarily contract concerns centered on as it was merely related to morbidity and life anticipation. World Health Organization (WHO) defines it as “the individual's perception of their life in the context of the culture and value systems in which they live and relate to their goals, expectations, standards, and concerns” (8). Quality of life may be a broad-ranging concept influenced in a complex way by the person's physical wellbeing, mental state, level of autonomy, social connections, and relationship to notable highlights of their environment (9). The most widely reported challenges that have been obliterating the quality of life of women living with obstetric fistulas are the psychosocial results of the fistula, shame, physical segregation, cessation of sexual relationships, and misfortune of status (10–12).

Most of the studies conducted on obstetric fistula are qualitative studies with a few quantitative studies (13–20). Most studies have focused on limited aspects of the total experience; such as the incidence and measurement of postpartum depression. In Ethiopia, only a few studies dealt with measuring the quality of life of women living with obstetric fistula and the factors influencing it.

Assessing the quality of life of women living with obstetric fistulas in each component of their quality of life (physiological, psychological, social relationships, and environmental) and predicting factors are essential to evaluate and understand the holistic well-being of women with fistulas and important inputs for effective maternal health interventions. Hence, this study planned to evaluate the level of quality of life and identify predictive variables across four domains of quality of life and for the overall quality of life among women living with obstetric fistula in Ethiopia.

## Materials and methods

### Study area, design, and period

A cross-sectional study was conducted from April 01 to August 01, 2019, at five fistula treatment centers in Ethiopia: Jimma University Medical Center, Asella Hospital, Harar, Mettu, and Addis Ababa Hamlin fistula centers.

### Study population and inclusion criteria

The populations included in this study were all women with obstetric fistula (mostly vesicovaginal fistula cases). These included: women who were diagnosed with obstetric fistula and waiting for surgery; those after 1 week of surgery before their discharge; and those who were appointed after surgery for a checkup.

### Exclusion criteria

In this study, twenty-one fistula women who were not volunteered to participate, those with severe fistula with complications (infections or comorbidities), cases of fistula secondary to trauma/surgery, and who cannot be able to respond to the study were excluded from the study.

### Sample size and sampling technique

**Figure 1:**
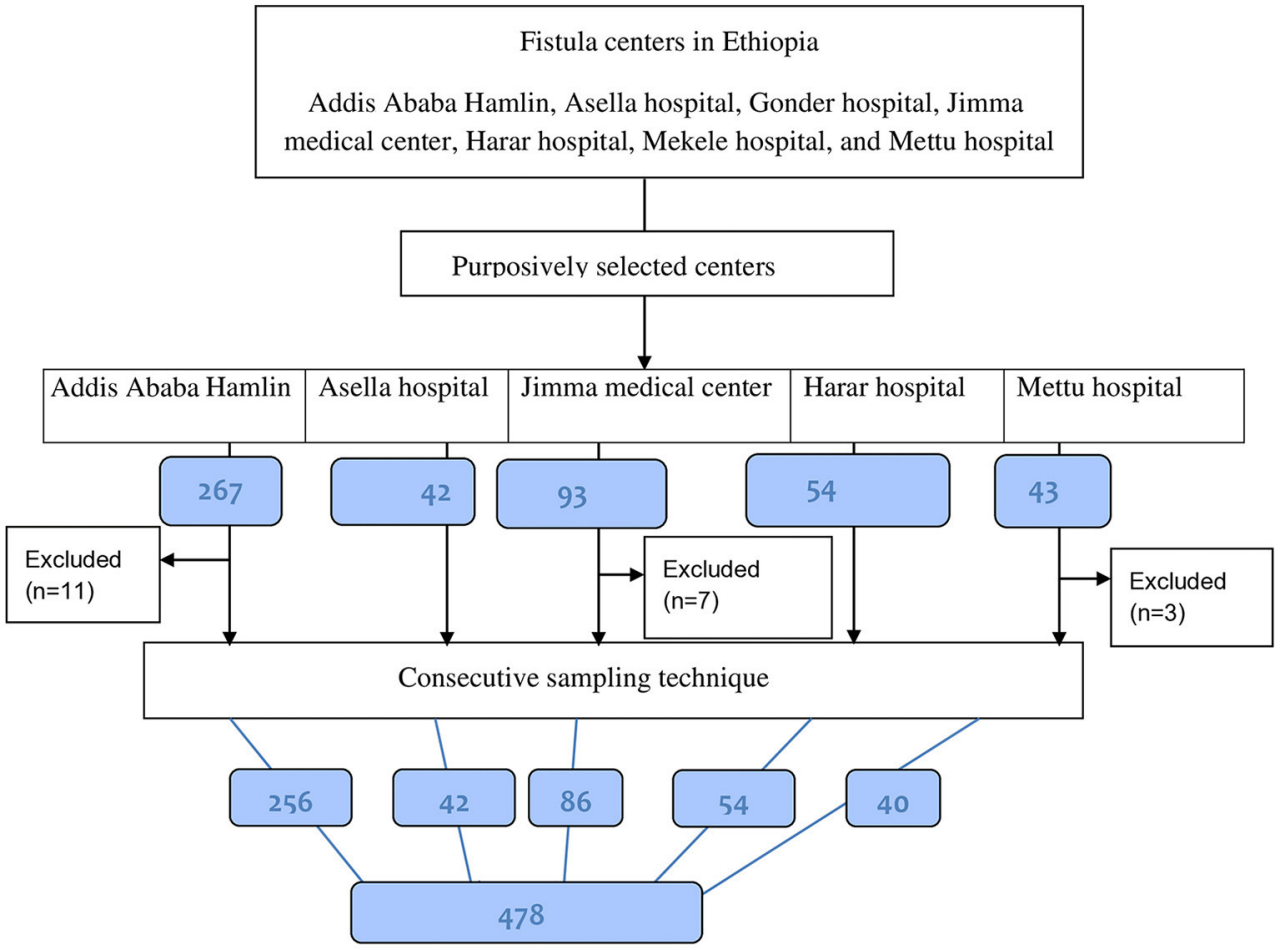
Diagrammatic representation of the sampling technique.

The sample size was estimated using the single population proportion formula. This was based on the assumptions [1.2% prevalence of obstetric fistula in Oromia Region (5); 95% confidence level, 1% precision, and 5% nonresponse rate]. Accordingly, 478 samples of women living with obstetric fistula were included in the study. The quotas of women consecutively selected during the study period from each study area were: 256 women from Addis Ababa Hamlin fistula center, 54 from Harar fistula center, 42 from Asella hospital, 40 from Mettu Hamlin fistula center, and 86 from Jimma University Medical Center (see Figure 1).

### Operational definitions

**Domain**: indicates the four health components of quality of life (21).

**Intention:** what someone plans to do (According to Merriam webster and Cambridge dictionary).

**High intention:** Scoring 31–40 on questions on intention to prevent obstetric fistula recurrence and classification was based on previous studies (16, 22, 23).

**Low intention**: Scoring 1–30 on questions on intention to prevent obstetric fistula recurrence (16, 22, 23).

**Knowledge:** It is measured based on twelve items of the knowledge of the risk factors questionnaire (24).

**Attitude:** Is the way that one considers and feels around something or the way one carries on with someone (22).

**Positive attitude:** Scoring 86–170 on attitude questions on obstetric fistula recurrence prevention (22).

**Negative attitude**: Scoring 1–85 on attitude questions on obstetric fistula recurrence prevention (22).

**Primary school**: Participants' educational status from grade one up to grade eight.

**Secondary and preparatory school**: participants' educational status from grade nine to grade twelve.

**Self Esteem:** It is someone's set consideration and sentiments, almost her/him worth and significance that's a worldwide positive or negative state of mind toward oneself (22).

**Time of care-seeking**: the time at which women with fistula started care-seeking.

### Data collection tool

The data collection tool was adapted from the WHO Quality of Life shortened version (WHOQOL-BREF). It has a four-domain structure and contains 26 items with five Likert scales for each variable. The four-domain scores indicate an individual's discernment of quality of life in each specific domain. The WHOQOL-BREF is based on a cross-culturally sensitive concept and found appropriate for use across different nationalities and confirmed to have good to excellent psychometric properties of reliability and validity. According to the world health organization quality of life assessment shortened version tool (WHOQOL-BREF), quality of life has four components or sections, commonly called domains. Of the 26 items/ facets of the WHOQOL-BREF tool, physical and environmental health domains each contained seven and eight facets respectively. The social relationships domain had three facets, and psychological health contained six facets. The remaining two items were examined separately and used to assess an individual's overall perception of quality of life and overall perception of their general health. Accordingly, the four domain scores denote an individual's perception of quality of life in each particular domain (21).

Other tools were also prepared from reviewing different articles and used for assessment of predicting factors such as:- attitudes of women with obstetric fistula toward obstetric fistula recurrence prevention with 34 items (five Likert scales) (16, 23), and Rosenberg's Self-Esteem Scale with 10 items (adapted, four Likert scales) (25). Attitudes toward prevention of fistula recurrence were scored from 1–85 to 86–170 on attitude questions on obstetric fistula recurrence prevention for negative and positive attitudes, respectively (22). Self-esteem is someone's set consideration and sentiments, almost her/him worth and significance that's a worldwide positive or negative state of mind toward oneself (22). According to Merriam-Webster and Cambridge dictionary definitions, the intention is what someone plans to do. Scoring 1–30 and above 31 on questions on intention to prevent obstetric fistula recurrence items were classified as low and high intention, respectively (16, 22, 23). Knowledge of women on the risk factors of fistula recurrence was measured based on twelve items of the knowledge of the risk factors questionnaire (24).

The tools were translated from the English language to local languages; retranslated back to English and pretested on 5% of the sample size of the study participants and found reliable with Cronbach's alpha value of (Quality of life = 0.83, attitude toward obstetric fistula recurrence prevention = 0.86, and self-esteem = 0.78).

### Data collection procedure

Before data collection, Jimma University's Institutional Review Board (IRB) approved the study (Ref.No: IRB 000281/2019). All participants signed an informed consent form before participation. Face-to-face interviews were conducted to collect data by five MSc Midwifery students and ten midwifery staff and supervised by five senior MSc Midwives.

### Data analysis

Data were entered into Epi data version 3.5.3 (Odense Denmark) using double data entry verification. Then the data were coded, cleaned, and exported to International Business Machines Corporation Statistical Product and Service Solutions (IBM SPSS) version 23 for further cleaning and analysis. Initially, exploratory data analysis was made and cleaned for missing values and outliers. Descriptive statistics were analyzed for socio-demographic and reproductive health-related factors. Some socio-demographic variables such as the age of respondents and distance to the nearest health facility on foot were categorized based on the data of respondents. Domain scores were computed for every four components/part of the quality of life and the scores were scaled in a positive heading (i.e., higher scores denote a higher quality of life) (26, 27). Two items were examined separately for women's overall perception of their quality of life and health based on the WHOQOL-BREF guideline (26, 27).

Item scores were computed for factors such as intention and attitude toward prevention of fistula recurrence, and self-esteem, and categorized as positive/negative attitudes and intentions, based on below half/ above half of each factor's item-total scores as cut-off scores. The significance level was announced at *P* < 0.05. The findings of the study were displayed utilizing tables, figures, and textual writings.

## Results

### Socio-demographic characteristics of participants

Among the 499 women with fistulas contacted for the interview, 478 were included in the study. Close to half (48.10%) of them were in the age group 25–34 (mean 28.90, SD ± 9.00) years. Three-fourths (75.50%) of the study participants were rural residents, and more than two-thirds (83.5%) of the women's occupation was unpaid employment. Two-thirds (66.30%) of the study participants were cannot read and write. More than half (60.00%) of them were in a married marital status while more than one-third (34.10%) of them were divorced or separated from their husbands, and another one-third (34.50%) have lived only with their husbands (see Table 1).

**Table 1:** Socio-demographic characteristics of women with obstetric fistula in Ethiopia, 2019 (*n* = 478).

**Factors**	**Categories**	***n* (%)**
Age at interview	15–24	137 (28.70)
	25–34	230 (48.10)
	35–44	80 (16.70)
	≥45	31 (6.50)
Mean age (±SD)	28.90 (±9.00)	
Residence	Urban	117 (24.50)
	Rural	361 (75.50)
Marital status	Never married	13 (2.70)
	Married	291 (60.90)
	Divorced/separated	163 (34.10)
	Widowed	11 (2.30)
Occupation	Paid works	79 (16.50)
	Unpaid works	399 (83.50)
Educational status	Can't read and write	317 (66.30)
	Read and write	35 (7.4)
	Primary schools	91 (19.00)
	Secondary and preparatory school	25 (5.20)
	College/university	10 (2.10)
Fathers educational status	Can't read and write	386 (80.80)
	Read and write	31 (6.50)
	Primary schools	12 (2.50)
	Secondary and preparatory school	10 (2.00)
	Don't know	39 (8.20)
Living with whom	Only with husband	165 (34.50)
	Only with husband and children	28 (5.90)
	Only with parents	147 (30.80)
	Relatives	36 (7.50)
	Only with children	16 (3.30)
	Alone	86 (18.00)

SD, standard deviation.

### Reproductive health histories of participants

One-fourth (26.80%) and above half (60.50%) of the study participants got married within age 10–14 and 15–19 years, respectively. The mean age at first pregnancy of the respondents was 17.80 ± 3.50) years. A large proportion (43.50%) of the respondents reported having encountered obstetric fistula between the age group 15–19 years with a mean age of 21.60 (SD ±7.00) years. Half (51.80%) of the women with obstetric fistula were primiparas. Most of them (83.70%) had stillbirths for the pregnancy to which they developed an obstetric fistula and 22.20% had a recurrence of obstetric fistula for third and more times. The mean of time lived with obstetric fistula was 5.02 ± 7.44 years (see Table 2).

**Table 2:** Reproductive health history of women with fistula in Ethiopia, 2019 (*n* = 478).

**Factors**	**Categories**	***n* (%)**
Age at first marriage	10–14	128 (26.80)
	15–19	289 (60.50)
	20–24	48 (10.00)
	≥25	13 (2.70)
Mean age at first marriage (±SD)	15.90 (±3.00)	
Age at first pregnancy	10–14	44 (9.20)
	15–19	322 (67.40)
	20–24	85 (17.80)
	≥25	27 (5.60)
Mean age at first pregnancy (±SD)	17.80 (±3.50)	
Number of parity	Primipara	248 (51.80)
	Multipara	159 (33.30)
	Grand-multipara	71 (14.90)
Age at the occurrence of fistula	10–14	34 (7.10)
	15–19	208 (43.50)
	20–24	98 (20.50)
	≥25	138 (28.90)
Types of fistula	Vesicovaginal	412 (86.19)
	Rectovaginal	47 (9.83)
	Combined	19 (3.98)
Mean age at occurrence of fistula (±SD)	21.60 (±7.00)	
Pregnancy at the occurrence of fistula	Planned	302 (63.20)
	Not planned	176 (36.80)
Attitude toward fistula prevention	Negative	267 (55.90)
	Positive	211 (44.10)
Intention toward fistula prevention	Negative	303 (63.40)
	Positive	175 (36.60)
Mean self-esteem(±SD)	24.52 ± 4.76	
Outcomes of labor for which fistula occurred	Live	78 (16.30)
	Stillbirth	400 (83.70)
Number of times fistula encountered	First time	247 (51.60)
	Second time	125 (26.20)
	Third time and more	106 (22.20)
Treatment status	Waiting for treatment	231 (48.30)
	Treated	247 (51.70)
Previous repair outcome	With no inconsistence	68 (27.50)
	With consistency	179 (72.50)
Mean for a time lived with obstetric fistula in years (±SD)	5.02 ± 7.44	
Time of care-seeking in weeks (±SD)	205 ± 87.63	
Place of care-seeking	Health facility	456 (95.40)
	Traditional healers	15 (3.10)
	*Others	7 (1.50)

^*^Stay at home.

### Quality of life of women with obstetric fistula

**Figure 2:**
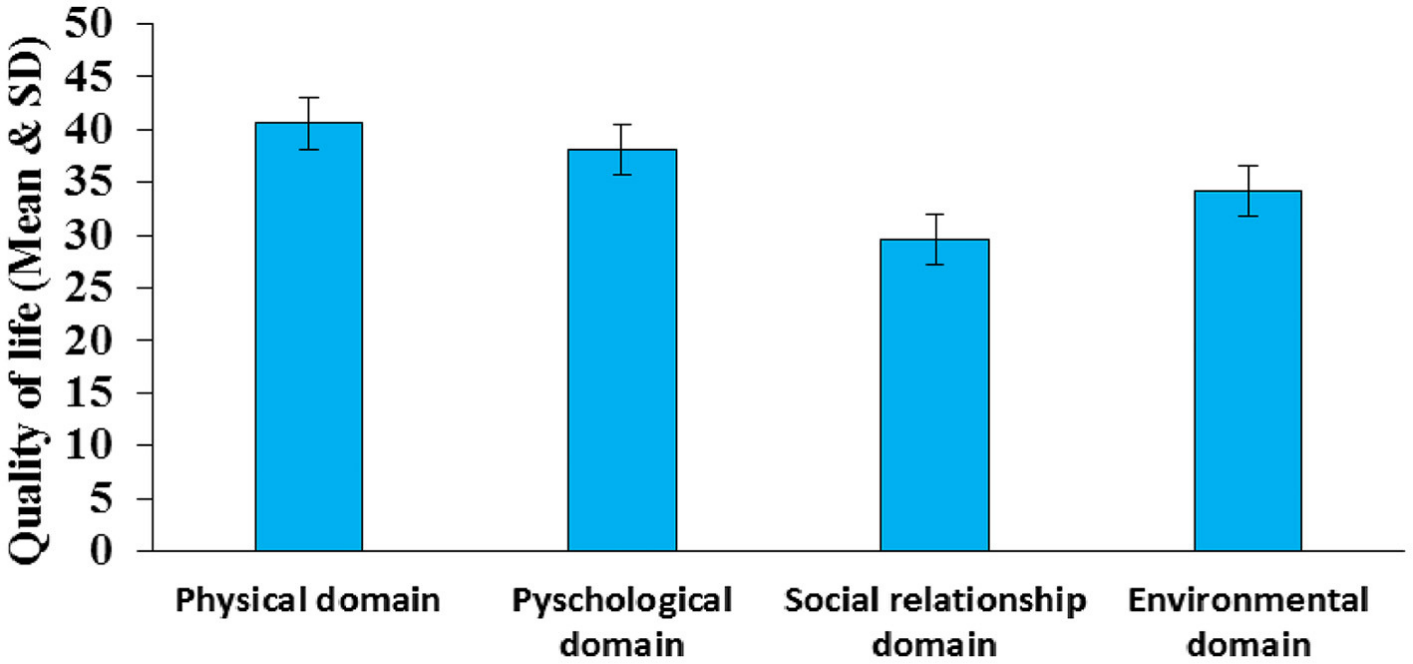
Mean scores of quality of life domains among women with obstetric fistula.

The mean levels of quality of life of women with obstetric fistula were found to be 40.59 ± 1.58 in the physical health domain, 38.10 ± 1.78 in the psychological domain, 34.21 ± 1.65 in the environmental health domain, and 29.59 ± 1.97 in the social relationship domain (see Figure 2).

In addition to the four domains of quality of life, we also measured the overall perceived quality of life and health status of the fistula women. The overall quality of life and satisfaction with health mean scores were 44.61 ± 3.99 and 33 ± 3.46 respectively. About 28.5% of the participants reported their overall quality of life as good, whereas 20.9% said that it was poor or very poor. About 23.6% said it was neither good nor poor, while the remaining 6.3% reported being very good. Regarding satisfaction with their health, 32.2% of the respondents replied that they were dissatisfied or very dissatisfied, 14.2% were neither satisfied nor dissatisfied, 17.4% were satisfied, and 4.8% were very satisfied.

### Predictors for the quality of life of women with obstetric fistula

A simple linear model was fitted for four domains of quality of life and predicting factors. Factors with *P*-value < 0.05 in the simple linear regression analysis were made eligible for multiple linear regression analysis.

Results of multiple linear regression indicated that: fistula repair outcomes without inconsistency, self-esteem, negative attitude toward prevention of fistula recurrence, waiting for treatment for fistula repair, and low intention on prevention of fistula recurrence were significant predictors in the physical domain quality of life. Variables such as women's fistula repair outcomes without inconsistency, self-esteem, negative attitude toward prevention of fistula recurrence, father education status at primary school, living with parents, time of care-seeking, and duration of living with fistula were significant predictors in the psychological domain. Living in rural, dead birth, self-esteem, and living only with parents and husbands were statistically significant predictors of social domain quality of life. Predictors such as rural residence, women's educational status at secondary school, self-esteem, negative attitude toward fistula recurrence prevention, time of care-seeking, and seeking care from a health facility were statistically significant predictors in the environmental domain (see Table 3).

**Table 3:** Predictors of quality of life domains among women with obstetric fistula in Ethiopia, 2019 (*n* = 478).

**Variables**	**Four domains of quality of life**												**Overall quality of life** ***R***^**2**^ = **0.21**		
	**Physical** ***R***^**2**^ = **0.27**			**Psychological** ***R***^**2**^ = **0.38**			**Social** ***R***^**2**^ = **0.24**			**Environ mental** ***R***^**2**^ = **0.35**			
	**Crude β (95% CI)**	**Adj unst β (95% CI)**	***P*-value**	**Crude β (95% CI)**	**Adj unst β (95% CI)**	***P*-value**	**Crude β (95% CI)**	**Adj unst β (95% CI)**	***P*-value**	**Crude β (95% CI)**	**Adj unst β (95% CI)**	***P*-value**	**Crude β (95% CI)**	**Adj unst β (95% CI)**	***P*-value**
Rural residence							−6.10 (−10.03, −2.16)^†^	−4.70 (−9.31, −0.09)*	0.046	−7.51 (−10.93, −4.09)^†^	−6.01 (−9.22, −2.79)*	< 0.001	−9.11 (−15.55, −2.68)^†^		0.006
Secondary school										2.88 (1.51,4.25)^†^	14.08 (3.67,24.48)*	0.008	3.32 (0.75,5.90)^†^		0.012
Dead birth							−10.53 (−15.08, −5.99)^†^	−5.18 (−9.86, −0.51)*	0.030						
With no inconsistence	6.44 (1.95, 10.93)^†^	5.18 (0.72, 9.64)*	0.023	8.09 (3.31, 12.87)^†^	5.86 (1.73, 9.99)*	0.006							11.37 (2.30, 19.75)^†^	8.32 (0.62, 16.02)*	0.034
Self-esteem	1.40 (1.08, 1.69)^†^	1.27 (0.96, 1.57*	< 0.001	1.91 (1.60, 2.22)^†^	1.79 (1.47, 2.11)*	< 0.001	1.37 (1.03, 1.71)^†^	1.07 (0.72, 1.43)*	< 0.001	1.57 (1.29, 1.85)^†^	1.27 (0.99, 1.55)*	< 0.001	2.51 (1.98, 3.10)^†^	2.06 (1.34, 2.79)*	< 0.001
Negative attitude	8.13 (5.03, 11.23)^†^	5.10 (1.86, 8.33)*	0.002	−9.28 (−12.58, −5.98)^†^	−6.43 (−9.60, −3.25)*	< 0.001				−5.24 (−8.23, −2.26)^†^	−5.13 (−7.97, −2.29)*	< 0.001			
Waiting treatment	−3.58 (−6.73, −0.43)^†^	−8.4 (−15.54, −1.10)*	0.024												
Low intention	6.35 (3.11, 9.58)^†^	4.73 (1.52, 7.93)*	0.004												
Fathers at primary education				1.75 (0.58, 2.91)^†^	12.45 (24.82, 0.08)*	0.049									
Living only with parents				1.05 (0.10, 1.99)^†^	4.94 (0.99, 8.90)*	0.014	2.41 (1.48, 3.34)^†^	5.50 (0.30, 10.69)*	0.038				3.44 (1.95, 4.93)^†^	2.91 (1.06, 4.76)*	0.002
Living only with husband							7.58 (4.05, 11.10)^†^	7.78 (2.01, 13.55)*	0.008						
Time of care-seeking				−0.01 (−0.02, −0.002)^†^	−0.01 (−0.02, −0.002)*	0.013				−0.013 (−0.02, −0.006)^†^	−0.009 (−0.015, −0.002)*	0.008			
Seek care from a health facility										8.07 (1.10, 15.03)^†^	11.77 (4.37, 19.17)*	0.002			
Time lived with fistula in years				−3.32 (−5.17, −1.48)^†^	−5.40 (−9.12, −1.68)*	0.005									

^†^Significant at p < 0.05, *significant at p < 0.001, < 0.01, and < 0.05, ^Υ^factors with no association, Adj unst β = adjusted unstandardized Beta, R^2^ = model fitness.

## Discussion

In this study, the mean scores for the quality of life of women with obstetric fistula were 40.59 ± 1.58 in the physical domain, 38.10 ± 1.78 in the psychological domain, 34.21 ± 1.65 in the environmental domain, and 29.59 ± 1.97 in the social relationship domain. Statistically significant predictors of the quality of life of women with fistula were: repair outcomes, self-esteem, negative attitude, and low intentions toward prevention of fistula recurrence, duration of living with fistula, treatment status, the outcome of labor, residence, women's and their father's educational status, living with whom, time of care-seeking, and a place where care was sought.

In our recent study, the mean scores for quality of life of women within the physical and psychological domains are consistent with a similar study conducted in Ethiopia but with lower mean scores within the social and environmental domains (28). This may indicate the continued social isolation experienced by victims. Similarly, there were higher psychological and social domain scores than in previous studies in Nigeria (29). There were also higher physical, psychological, and environmental domain scores in this study than in the previous study in India (30). This discrepancy may be because of differences in time of the study, sample size, and socio-demographic features of the study area from the previous studies. Similarly in this study, women's overall perception of the quality of life and health was lower than in a previous study conducted in Bangladesh (6). This may be due to the differences in the measurement tools used to estimate the quality of life in a recent study, sample sizes used, time of the study, and socio-demographic characteristics of the study areas.

Women whose repair outcome was with no urinary inconsistency had increased quality of life within the psychological and physical domains as well as in their overall quality of life. This is because women who are free from incontinence of urine and feces are more intact physically and stable psychologically. They are also more free from foul-smelling odors. This is consistent with a similar study, showing that remaining incontinent after surgical fistula repair diminishes women's quality of life as they remain incontinent and still experience social and economic difficulties, stigma, or emotional pain (31). A similar study in Africa shows that women with closed fistula and with no incontinence have a better quality of life than those with proceeding incontinence (3). Another similar study also found a consistent premise that women with incontinence after repair have a lower quality of life scores (32).

This study showed for a unit increase in self-esteem there were increased means scores across the four domains of quality of life and overall quality of life. This is because women who have high self-esteem may also have high physical, psychological, social, environmental, and overall quality of life. High quality of life in the health domain is found to be associated with high self-esteem while the low quality of life is correlated with low self-esteem. This is in line with a similar study in Nigeria, which shows that high self-esteem is found to be associated with a decrease in the rate of depression for women living with vesicovaginal fistula (33).

Women with fistula who had a negative attitude toward the prevention of fistula recurrence had a lower psychological and environmental quality of life. This is maybe that women who have a negative attitude toward obstetric fistula prevention may not obey and follow preventive measures and fit poorly psychologically. This is in agreement with a study conducted in Benin which revealed that more than half of women living with fistula who had a negative attitude toward fistula prevention have felt that hospital delivery is not an obstetric fistula preventive measure (34). A similar study in Northern Nigeria also supports this premise that women living with a fistula who were not healthy psychologically have no willingness to use contraceptive methods to prevent the recurrence of the fistula by delaying pregnancy (35).

Women with a low intention and negative attitude toward the prevention of fistula recurrence had increased physical health domain scores of quality of life. This might be because women who have already been repaired and cured physically may need fewer prevention measures. Similarly, women who were waiting for treatment had a decreased physical quality of life by eight. This may be because women who are not repaired and waiting for the repair of urinary inconsistencies may have a lower quality of life than those who are repaired. This is compatible with a previous study in Ethiopia, which indicates that a larger part of women felt an emotional sensation of alleviation and bliss taken after repair that maximizes their quality of life (17).

In this study, women whose fathers' educational status was at primary school had an increased psychological quality of life than those whose fathers cannot read and write. This may be because those whose fathers are more educated may get understood easily their fistula condition; get more support, psychological reassurance, and encouragement. This is supported by a previous study showing that women whose fathers and spouses were knowledgeable about their fistula and treatment have got financial and psychosocial support and feel better (36). For a unit increase in years at a time of living with obstetric fistula, the psychological quality of life was decreased by five. This is because as the time of living with fistula conditions increases, women continue to experience different consequences of fistula with long-term emotional effects that in turn affect their psychological quality of life. This is in harmony with previous studies (37, 38).

Women who were rural residents had a decreased quality of life within the environmental and social components of quality of life by more than five. This is because women who live in rural have poor access to hygiene-keeping resources and they are more stigmatized by society than urban residents. However, women who were educated to the secondary school level had an increased quality of life in the environmental domain by fourteen. This might be because women who are more educated may have more knowledge on how to keep their personal and environmental hygiene than those who were not educated. This is congruent with a study in Iran, which shows that women who had college degrees have a higher quality of life scores than those who were not educated (39).

In this study, women who have been living with their parents had an increased quality of life in the psychological and social domains as well as in their overall quality of life. Similarly, women who had been living with their husbands had an increased quality of life in the social relationship domain of quality of life. This may be because women who are living with their parents and husbands can get psychological and other essential support. Therefore, they may have better social relationships. This is consistent with the previous study reports that having good relationships with their partners and family members contributes to a higher quality of life for women living with obstetric fistula (40, 41). The social relationship domain of quality of life of women with a stillbirth outcome was decreased five times more than those who had a live birth outcome. This is because women with obstetric fistula who have a live birth have a higher social acceptance and respect than those who have a stillbirth. Contrary to this, a study conducted in Bangladesh depicts no differences in the quality of life mean scores for women with fistula whether they had living children or not (6).

With a unit increase in time in the weeks before starting care seeking after obstetric fistula, that is delay in accessing surgical care, the psychological and environmental quality of life was decreased. This shows that as more times in weeks have elapsed before starting care for fistula repair, women remain untreated and continue experiencing urinary and fecal inconsistencies and related sequelae of the fistula. This then affects their psychological and environmental quality of life. A previous study also collaborates with this that the duration of incontinence of more than 5 years is found to negatively relate to the environmental quality of life (10, 38). Seeking care from health facilities increased environmental health quality of life, i.e., for women who sought care from health facilities, environmental quality of life increased by 12. This might be when women seek care from a health facility, they get skilled obstetric care, repair, hygienic care, and supports that increases their environmental health domain. This is congruous with previous studies which show that having care at a health institution with skilled attendants increases the quality of health care services. Such care in turn improves the overall health of women with fistulas (42, 43).

The strengths of this study are: First, it is an original study that assessed the quality of life and identified new predictors affecting the quality of life of women living with obstetric fistulas across four domains of their quality of life and the overall quality of life; using the most reliable and validated WHOQOL-BREF tool. Second, it included a larger number of study participants.

The limitation of this study is: It included women with obstetric fistulas found only in five fistula treatment centers and used a consecutive sampling technique.

## Conclusions

In this study, the quality of life of women living with obstetric fistula was low. Repair outcome, duration lived with fistula, self-esteem, attitude, and intention toward prevention of fistula recurrence, treatment status, outcome of labor, residence, women's and father's educational status, time of care-seeking, a place where care sought, and living with whom were significant predictors of quality of life. Interventions targeted at the improvement in the quality of life of women living with obstetric fistulas should address these factors for restoring women's holistic health and dignity. Policy-makers should emphasize in prioritizing surgical care services and awareness creation programs among women with fistulas. Healthcare practitioners should treat and contact such women empathically for the restoration of their impaired quality of life and dignity. Furthermore, this study is an important inputs of information for effective maternal health interventions.

## Data availability statement

The original contributions presented in the study are included in the article/supplementary material, further inquiries can be directed to the corresponding author.

## Ethics statement

The studies involving human participants were reviewed and approved by Jimma University's Institutional Review Board. Written informed consent to participate in this study was provided by the participants' legal guardian/next of kin.

## Author contributions

BFH and ZBK conceived the concept. BFH designed, implemented, supervised the fieldwork, analyzed the data, and wrote up the manuscript. ZBK and LSD were involved in the design of the study, data analysis, and critically revised the manuscript. All authors critically read and approved the submission of the final version of the manuscript for publication.

## Conflict of interest

The authors declare that the research was conducted in the absence of any commercial or financial relationships that could be construed as a potential conflict of interest.

## Publisher's note

All claims expressed in this article are solely those of the authors and do not necessarily represent those of their affiliated organizations, or those of the publisher, the editors and the reviewers. Any product that may be evaluated in this article, or claim that may be made by its manufacturer, is not guaranteed or endorsed by the publisher.

## References

[1] Tebeu PM Fomulu JN Khaddaj S de Bernis L Delvaux T Rochat CH. Risk factors for obstetric fistula: a clinical review. Int Urogynecol J. (2012) 23:387–94. 10.1007/s00192-011-1622-x 22143450 PMC3305871

[2] Polan ML Sleemi A Bedane MM Lozo S Morgan MA. Obstetric Fistula. Essential Surgery: Disease Control Priorities, 3rd Edn. Washington, DC: The International Bank for Reconstruction and Development/The World Bank (2015). 10.1596/978-1-4648-0346-8_ch6

[3] Landry E Frajzyngier V Ruminjo J Asiimwe F Barry TH Bello A. Profiles and experiences of women undergoing genital fistula repair: findings from five countries. Glob Public Health. (2013) 8:926–42. 10.1080/17441692.2013.824018 23947903 PMC3805436

[4] Jokhio AH Rizvi RM Rizvi J MacArthur C. Prevalence of obstetric fistula: a population-based study in rural Pakistan. BJOG. (2014) 121:1039–46. 10.1111/1471-0528.12739 24684695

[5] Biadgilign S Lakew Y Reda AA Deribe K. A population based survey in Ethiopia using questionnaire as proxy to estimate obstetric fistula prevalence: results from demographic and health survey. Reprod Health. (2013) 10:14. 10.1186/1742-4755-10-14 23432944 PMC3598195

[6] Walker S Ambauen-Berger B Saha S Akhter S. Quality of life among women in Bangladesh following ileal conduit urinary diversion operations for irreparable vesicovaginal fistula and bladder exstrophy: observational study. BJOG Int J Obstet Gynaecol. (2018) 125:616–22. 10.1111/1471-0528.14721 28467691

[7] Jungari S Chauhan BG. Obstetric fistula in Assam, India: a neglected cause of maternal morbidities and mortality. Healthcare Low Resour Settings. (2015) 3:4663. 10.4081/hls.2015.4663

[8] Gholami A Jahromi LM Zarei E Dehghan A. Application of WHOQOL-BREF in measuring quality of life in health-care staff. Int J Prevent Med. (2013) 4:809. 10.1023/a:1016351406336 24049600 PMC3775221

[9] Berlim MT Fleck M. “Quality of life”: a brand new concept for research and practice in psychiatry. Rev Brasil Psiquiat. (2003) 25:249–52. 10.1590/S1516-44462003000400013 15328553

[10] Imoto A Matsuyama A Ambauen-Berger B Honda S. Health-related quality of life among women in rural Bangladesh after surgical repair of obstetric fistula. Int J Gynaecol Obstet Off Organ Int Federat Gynaecol Obstet. (2015) 130:79–83. 10.1016/j.ijgo.2015.01.018 25935472

[11] Degge HM Laurenson M Dumbili EW Hayter M. Reflections on identity: narratives of obstetric fistula survivors in North Central Nigeria. Qual Health Res. (2019) 30:1049732319877855. 10.1177/1049732319877855 31578929

[12] Cowgill KD Bishop J Norgaard AK Rubens CE Gravett MG. Obstetric fistula in low-resource countries: an under-valued and under-studied problem—systematic review of its incidence, prevalence, and association with stillbirth. BMC Preg Childbirth. (2015) 15:193. 10.1186/s12884-015-0592-2 26306705 PMC4550077

[13] Wall LL Belay S Haregot T Dukes J Berhan E Abreha M. case–control study of the risk factors for obstetric fistula in Tigray, Ethiopia. Int Urogynecol J. (2017) 28:1817–24. 10.1007/s00192-017-3368-6 28550462

[14] Tunçalp Ö Tripathi V Landry E Stanton CK Ahmed S. Measuring the incidence and prevalence of obstetric fistula: approaches, needs and recommendations. Bull World Health Organ. (2014) 93:60–2. 10.2471/BLT.14.141473 25558110 PMC4271685

[15] Maheu-Giroux M Filippi V Samadoulougou S Castro MC Maulet N Meda N. Prevalence of symptoms of vaginal fistula in 19 Sub-Saharan Africa countries: a meta-analysis of national household survey data. Lancet Global Health. (2015) 3:e271–8. 10.1016/S2214-109X(14)70348-1 25889469

[16] Gebresilase YT A. qualitative study of the experience of obstetric fistula survivors in addis Ababa, Ethiopia. Int J Women's Health. (2014) 6:1033. 10.2147/IJWH.S68382 25525395 PMC4266262

[17] Donnelly K Oliveras E Tilahun Y Belachew M Asnake M. Quality of life of Ethiopian women after fistula repair: implications on rehabilitation and social reintegration policy and programming. Cult Health Sex. (2015) 17:150–64. 10.1080/13691058.2014.964320 25317830

[18] Barageine JK Tumwesigye NM Byamugisha JK Almroth L Faxelid E. Risk factors for obstetric fistula in Western Uganda: a case control study. PLoS ONE. (2014) 9:e112299. 10.1371/journal.pone.0112299 25401756 PMC4234404

[19] Ballard K Ayenachew F Wright J Atnafu H. Prevalence of obstetric fistula and symptomatic pelvic organ prolapse in rural Ethiopia. Int Urogynecol J. (2016) 27:1063–7. 10.1007/s00192-015-2933-0 26755052

[20] Ahmed S Tunçalp Ö. Burden of obstetric fistula: from measurement to action. Lancet Global Health. (2015) 3:e243–4. 10.1016/S2214-109X(15)70105-1 25889463

[21] Webster J Nicholas C Velacott C Cridland N Fawcett L. Validation of the WHOQOL-BREF among women following childbirth. Aust N Z J Obstet Gynaecol. (2010) 50:132–7. 10.1111/j.1479-828X.2009.01131.x 20522068

[22] Nambala N Mweemba P Labib M. Women's intention to prevent vesico vaginal fistula recurrence in two repair centres in Zambia. Med J Zambia. (2012) 39:54–8. 10.55320/mjz.39.2.418

[23] Lufumpa E Doos L Lindenmeyer A. Barriers and facilitators to preventive interventions for the development of obstetric fistulas among women in Sub-Saharan Africa: a systematic review. BMC Preg Childbirth. (2018) 18:155. 10.1186/s12884-018-1787-0 29747604 PMC5946543

[24] Banke-Thomas AO Kouraogo SF Siribie A Taddese HB Mueller JE. Knowledge of obstetric fistula prevention amongst young women in Urban and Rural Burkina Faso: a cross-sectional study. PLoS ONE. (2013) 8:e85921. 10.1371/journal.pone.0085921 24392032 PMC3877393

[25] Rosenberg M. Society and the Adolescent Self-Image. Princeton: Princeton University Press (2015).

[26] Skevington SM Lotfy M O'Connell KA. The world health organization's WHOQOL-BREF quality of life assessment: psychometric properties and results of the international field trial. A report from the whoqol group. Qual Life Res. (2004) 13:299–310. 10.1023/B:QURE.0000018486.91360.00 15085902

[27] Organization WH. WHOQOL-BREF: Introduction, Administration, Scoring and Generic Version of the Assessment: Field Trial Version, December 1996. Geneva: World Health Organization (1996).

[28] Matiwos B Tesfaw G Belete A Angaw DA Shumet SJB. Quality of life and associated factors among women with obstetric fistula in Ethiopia. BMC Womens Health. (2021) 21:1–9. 10.1186/s12905-021-01458-3 34454486 PMC8403383

[29] Umoiyoho A Inyang-Etoh E Abah G Abasiattai A Akaiso O. Quality of life following successful repair of vesicovaginal fistula in Nigeria. Rural Remote Health. (2011) 11:1734. 10.22605/RRH1734 21905761

[30] Singh V Jhanwar A Mehrotra S Paul S Sinha R. A comparison of quality of life before and after successful repair of genitourinary fistula: is there improvement across all the domains of WHOQOL-BREF questionnaire? Afr J Urol. (2015) 21:230–4. 10.1016/j.afju.2015.06.003

[31] Mafo Degge H Hayter M Laurenson M. An integrative review on women living with obstetric fistula and after treatment experiences. J Clin Nurs. (2017) 26:1445–57. 10.1111/jocn.13590 27680693

[32] Kopp D Tang J Bengtson A Chi B Chipungu E Moyo M. Continence, quality of life and depression following surgical repair of obstetric vesicovaginal fistula: a cohort study. BJOG Int J Obst Gynaecol. (2019) 126:926–34. 10.1111/1471-0528.15546 30461170 PMC6510632

[33] Takai MG. Depressive disorder, quality of life, coping and self esteem among patients with vesicovaginal fistula seen in kano: prevalence and sociodemographic correlates [dissertation]. Aminu Kano Teaching Hospital(Niger), Bayero University (2015).

[34] Gharoro E Agholor K. Aspects of psychosocial problems of patients with vesico-vaginal fistula. J Obstet Gynaecol J Instit Obstet Gynaecol. (2009) 29:644–7. 10.1080/01443610903100609 19757273

[35] Uchendu O Adeoti H Adeyera O Olabumuyi O. After obstetric fistula repair; willingness of women in northern Nigeria to use family planning. J Obstet Gynaecol. (2019) 39:313–8. 10.1080/01443615.2018.1514591 30428739

[36] Warren CE Sripad P Mwangi A Ndwiga C Liambila W Bellows B. “Sickness of Shame”: Investigating Challenges and Resilience among Women Living with Obstetric Fistula in Kenya. In: Global Perspectives on Women's Sexual and Reproductive Health across the Lifecourse. New York, NY: Springer (2018), 91–109. 10.1007/978-3-319-60417-6_6

[37] Bashah DT Worku AG Mengistu MY. Consequences of obstetric fistula in Sub Sahara African countries, from patients' perspective: a systematic review of qualitative studies. BMC Womens Health. (2018) 18:106. 10.1186/s12905-018-0605-1 29925358 PMC6011512

[38] El Ayadi AM Barageine J Korn A Kakaire O Turan J Obore S. Trajectories of women's physical and psychosocial health following obstetric fistula repair in Uganda: a longitudinal study. Trop Med Int Health. (2019) 24:53–64. 10.1111/tmi.13178 30372572 PMC6324987

[39] Rezaei N Azadi A Zargousi R Sadoughi Z Tavalaee Z Rezayati M. Maternal health-related quality of life and its predicting factors in the postpartum period in Iran. Scientifica. (2016) 2016:8542147. 10.1155/2016/8542147 27022506 PMC4789062

[40] Kakembo S Atuhairwe C Taremwa IM. Quality of life among obstetric fistula patients at kitovu mission hospital: a health facility-based cross-sectional study in Masaka District, Uganda. Obstet Gynecol Int. (2020) 2020:7953915. 10.1155/2020/7953915 32528539 PMC7262733

[41] Dennis AC Wilson SM Mosha MV Masenga GG Sikkema KJ Terroso KE. Experiences of social support among women presenting for obstetric fistula repair surgery in Tanzania. Int J Women's Health. (2016) 8:429. 10.2147/IJWH.S110202 27660492 PMC5019876

[42] Mselle LT Kohi TW. Healthcare access and quality of birth care: narratives of women living with obstetric fistula in Rural Tanzania. Reprod Health. (2016) 13:1–9. 10.1186/s12978-016-0189-x 27449061 PMC4957307

[43] Mselle LT Moland KM Mvungi A Evjen-Olsen B Kohi TW. Why give birth in health facility? Users' and providers' accounts of poor quality of birth care in Tanzania. BMC Health Serv Res. (2013) 13:1–12. 10.1186/1472-6963-13-174 23663299 PMC3654954

